# Exploring the Ethical Dimensions of Care Dependency in Older People: A Transpersonal View

**DOI:** 10.1111/scs.70127

**Published:** 2025-10-03

**Authors:** Ana‐María Porcel‐Gálvez, Enrique‐Octavio Iñiguez‐Castro, Gloria Martínez‐Lacovic, Regina Allande‐Cussó

**Affiliations:** ^1^ Research Group CTS 1050 Complex Care, Chronicity and Health Outcomes, Institute of Biomedicine of Seville (IBiS) University of Seville Seville Spain; ^2^ Nursing Department, Faculty of Nursing, Physiotherapy and Podiatry University of Seville Seville Spain

**Keywords:** care dependency, concept analysis, ethics, older adults, transpersonal nursing

## Abstract

**Introduction:**

Care dependency in older adults is a growing global concern, driven by population ageing and the increasing prevalence of chronic conditions. Beyond its clinical implications, care dependency involves ethical, cultural, and transpersonal dimensions that shape the lived experiences of individuals and caregivers. This study aims to refine the concept of care dependency through an explicitly transpersonal and culturally sensitive lens, informing practice, education, and policy.

**Methods:**

Following Walker and Avant's concept analysis framework, a systematic review of the literature was conducted. Key attributes, antecedents, and consequences were identified through an iterative process of data synthesis and theoretical reflection, including the development of model, borderline, and contrary cases to explicitly illustrate conceptual application.

**Results:**

The analysis identified four defining attributes: loss of autonomy; erosion of identity and self‐image; biographical rupture; and negative coping. Antecedents include physiological ageing, chronic degenerative disease, cognitive decline, and psychosocial/cultural factors that constrain autonomy. Consequences extend beyond functional limitations, affecting emotional well‐being and generating ethical and context‐specific decisions. The cases demonstrate a continuum from full dependency to complete autonomy and make visible how context and culture shape these attributes.

**Conclusions:**

Care dependency emerges as a multidimensional, ethically situated phenomenon, shaped by relationships, culture, and context. The proposed conceptual framework offers practical guidance for applying a transpersonal approach, prioritizing mutual presence, active listening, cultural attunement, and shared decision‐making to preserve dignity, meaning, and connection in daily care. Future research should explore the framework's applicability through qualitative studies in diverse settings to translate it into actionable, ethically grounded tools and interventions.

## 1 Introduction

In broad terms, care dependency is defined as a subjective need for support to compensate for a deficit in care [1]. Increased longevity does not inherently correspond to an improved quality of life. On the contrary, ageing is often accompanied by greater vulnerability, with individuals facing a heightened risk of social exclusion or dependency [2, 3].

The growing population of older, dependent people around the world demands an increase in the formulation of social and health policies, as well as an increase in research. Considering aspects such as culture, religion, or gender would give this topic an ethical and transpersonal approach that would enable a better understanding of the phenomenon [4].

From a care perspective, the framework of Person‐Centred Care (PCC) has evolved to address the complexities of providing holistic and individualised care [5]. Balint introduced the initial ideas of person‐centred medicine in the 1970s and emphasised the importance of the doctor–patient relationship and the need for a humanistic approach to healthcare [6]. A few years later, Carl Rogers advocated a person‐centred approach to health care within humanistic psychology [7]. As early as 2006, McCormack and McCance developed a comprehensive nursing framework integrating individualised care, autonomy, and respect for personal preferences, which they named the Person‐Centred Care Framework [8]. This framework is structured around key interrelated constructs, including prerequisites of the healthcare professional (such as their values, skills, and commitment), the care environment (which supports person‐centred practices), and person‐centred processes (involving shared decision‐making, engagement, and holistic care). It highlights the dynamic nature of care interactions and the necessity of fostering meaningful relationships to enhance patient experiences and outcomes [8].

From an ethical perspective, and based on Kant's categorical imperative, every human being, under their dignity, deserves to be treated with consideration and respect, as an end in themselves rather than merely as a means [9]. Furthermore, Heidegger's concept of *being‐in‐the‐world* highlights the relational nature of human existence, suggesting that individuals are defined by their interactions with others and their surroundings. This notion emphasizes that human beings do not exist in isolation but are always embedded within a socio‐cultural and historical context that shapes their experiences and understanding of the world [10].

In the same way, the ethical obligations of beneficence, autonomy, and justice in the care administered to dependent people are born. Thus, caring itself intrinsically relates to the commitment to protection and respect for human dignity and ethical principles [11]. The ethics of care is an ethics of responsibility of virtue, since the attitudes and behaviours will underpin the relationships between the caregiver and the person being cared for [11]. Caring is also based on an ethic of justice as it allows society to be reorganised according to individual needs [12]. Therefore, nursing care, from its most fundamental perspective, is linked to the ethical consideration of others, who may position themselves as vulnerable [13].

When the term dependency in older people is used in the context of care, there is a lack of clarity about its meaning. Sometimes the definition focuses only on the older person's ability to function without assistance, while in others, it could also allude to their lack of decision‐making capacity [14]. Furthermore, it seems to be the factor most frequently associated with adverse events in the clinical setting [15]. On the other hand, two other concepts should be considered as different statuses of care dependency: vulnerability, which refers to a heightened susceptibility to physical, psychological, or social harm, and frailty, which is a clinical syndrome involving a decline in physiological reserves and resilience, increasing the risk of adverse health outcomes [16, 17].

Care dependency entails the need for care by another person, and it is legitimate to think that a relationship is established between the caregiver and the person being cared for, which must be based on mutual trust, ethical values, and respect [18]. The concept of transpersonal care in nursing emphasizes the importance of integrating spiritual and intersubjective dimensions into healthcare interactions. It regards patients as unique, multidimensional beings and calls on nurses to broaden their understanding of both self and others to enhance autonomy and individuality in care encounters. From this perspective, the mental patterns and intentionality of both caregiver and recipient can influence care outcomes, opening possibilities for innovative approaches to planning, delivering, and evaluating nursing interventions [19].

Within nursing science, the transpersonal perspective is deeply rooted in established theoretical frameworks such as Watson's Theory of Human Caring and Eriksson's Caritative Caring Theory. Watson emphasizes the centrality of human‐to‐human connection, intentional presence, and the creation of a healing environment that supports autonomy, dignity, and holistic well‐being [20]. Similarly, Eriksson highlights the alleviation of suffering and the fostering of meaningful relationships as core elements of caring, with a strong ethical foundation in love, mercy, and compassion [21]. Together, these theories provide a practical and philosophical grounding for understanding care dependency as a multidimensional human experience and for guiding nursing interventions that address not only physical needs but also emotional, relational, and spiritual dimensions.

Transpersonal care becomes important here because it represents a holistic and humanistic approach to caring, emphasizing the deep connection between the caregiver and the care recipient beyond physical needs [20]. Grounded in Jean Watson's Theory of Human Caring, this perspective highlights the importance of empathy, presence, and a mutual commitment to human dignity. Transpersonal care recognizes that the experience of dependency in older people is not merely a functional decline but also a profoundly existential and emotional state. In this context, the caring relationship becomes a meaningful exchange that seeks to preserve the individual's sense of identity, autonomy, and well‐being despite their dependency [4]. It is also necessary to highlight the possible vulnerable situation experienced by the older people cared for, given that they potentially could have a lack of functional or cognitive autonomy as well as social or family relationships [22].

On the other hand, care practices imply great complexity, being linked to multiple cultural, social, institutional, economic, regulatory, and religious aspects, among others. In this sense, in the current situation of global migration, there are cultural differences in the profile of older people, which require an understanding of the concept not only from the etymological and physiological perspective but also from the ethical and transpersonal dimensions [23].

In this sense, this study aims to clarify the concept of care dependency in older people from an ethical and transpersonal perspective.

## 2 Material and Methods

Analysis of the concept through an integrative review of the literature, taking the framework of Walker and Avant's eight‐step procedure of concept analysis, was carried out [24].

Concept analysis is a procedure used to analyse and systematise the concepts of a discipline, obtaining clear, well‐defined, and common concepts that facilitate the development of theories and models related to it [24]. Following Walker and Avant's framework, the defining attributes were refined to the minimum essential elements required to distinguish the concept from others. Walker and Avant state that concept analysis is a way to build theory, which provides the opportunity to explain and describe phenomena of interest to nursing practice and involves 8 stages: (1) Selecting a concept; (2) Determining the aim and purpose of the analysis; (3) Identifying the uses (definitions) of the concept; (4) Determining defining attributes; (5) Identifying a model case; (6) Identifying borderline and contrary cases; (7) Identifying the antecedents and consequences of the concept; and (8) Defining the empirical referents. The inclusion of model, borderline, and contrary cases is a critical phase in the Walker and Avant methodology, as it allows for a deeper understanding of the concept by illustrating its presence, partial manifestation, or absence in real‐world scenarios. The model case represents a clear and unquestionable example of the concept, embodying all its defining attributes. In contrast, the contrary case serves as an example where the concept is absent, helping to delineate its boundaries. The borderline case, meanwhile, presents a situation in which some, but not all, defining attributes are present, highlighting areas of conceptual ambiguity [24].

### 2.1 Review Question

Initially, the PEO (Population, Exposure, Outcomes) question was identified: the first element of the strategy, (P), refers to older people, (E) care dependency, and (O) concept, attributes or characteristics, antecedents, and consequences from an ethical and transpersonal perspective. The aim of this study is not to compare it with any type of intervention or with similar concepts either. Thus, it was decided to pose an open question, typical of the integrative review, which could be answered with the selected methodology: What are the concept, the attributes or characteristics, the antecedents, and consequences of care dependency in older people from an ethical and transpersonal perspective?

### 2.2 Inclusion and Exclusion Criteria

- Inclusion criteria: (1) Articles that address topics related to the care of elderly people in situations of care dependency. (2) That also describe one or more of the attributes, antecedents, and consequences that characterise the concept. (3) And published in Spanish, English, or Portuguese. (4) No limit was placed on the year of publication, and an effort was made to describe the concept comprehensively.
- Exclusion criteria: studies related to dependence or abuse of opiates, drugs, and medications. Paediatric, adolescent, and/or young adult study population, and/or frailty and vulnerability studies, and/or dealing with care dependency without an ethical perspective. Access to the full text is not allowed without affiliation or year of publication.

For this study, care dependency was operationally defined as the presence of functional and/or cognitive limitations requiring partial or total assistance with basic activities of daily living [1]. While frailty, vulnerability, and social frailty share certain attributes with care dependency, such as vulnerability and the risk of social isolation, they do not inherently imply a continuous need for care. Vulnerability, as analyzed by Purdy, reflects the capacity to reorganize and engage in, or be selected for, interventions promoting a healthy trajectory, and may be present in both frailty and care dependency [25]. Frailty and functional limitations in older adults may result in a loss of independence [26], whereas social frailty primarily has psychosocial antecedents and attributes and does not necessarily entail ongoing assistance with basic activities of daily living [26]. In this sense, to ensure conceptual coherence, studies addressing frailty or vulnerability without a direct link to care dependency were excluded. This delineation remains a focus of our group's research and will inform future publications aimed at clarifying these concepts in nursing practice.

Studies were considered to have an ethical perspective if they explicitly or implicitly addressed values such as autonomy, dignity, beneficence, or justice in the context of care dependency. Qualitative works exploring these aspects were included even if ethical terminology was not explicitly used.

### 2.3 Search Strategy

The search for articles was carried out between March 2021 and December 2022, respecting the criteria and particularities of the following databases: SCOPUS, Cumulative Index of Nursing and Allied Health Literature (CINAHL), National Library of Medicine (MEDLINE via PubMed), and Web of Science (WOS). Grey literature was excluded, as the analysis focused solely on peer‐reviewed scientific literature to ensure methodological rigor and analytical consistency.

Grey literature and editorials were excluded to ensure methodological rigor and consistency.

Two searches were performed. The first search included the following terms: [(“dependency”) AND (caring OR care) AND (nursing OR nurse) NOT (“frail elderly” OR “vulnerability”) AND ethic]. The second search excluded the ethical terms to obtain more results, [(“dependency”) AND (nursing OR nurse) AND (caring OR care) NOT (“frail elderly” OR “vulnerability”)]. The terms related to frailty were excluded considering that it does not inherently entail a situation of care dependency [13].

### 2.4 Selection of Studies

**FIGURE 1:**
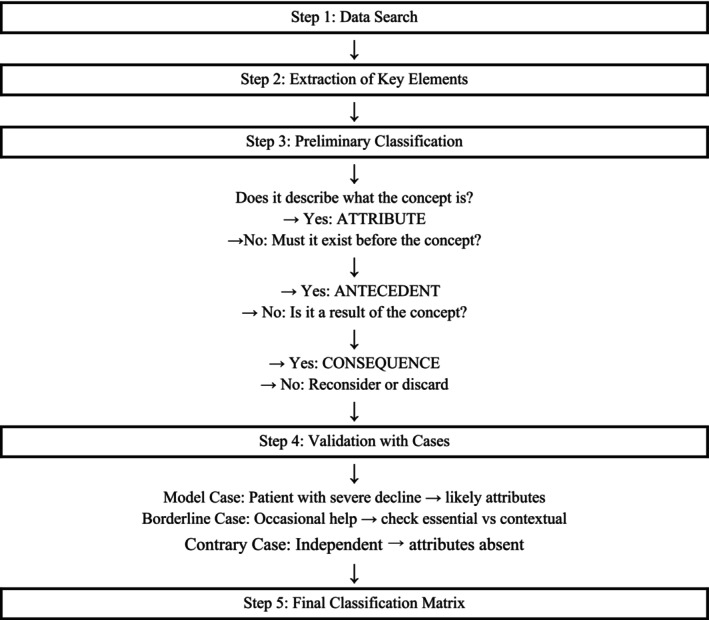
Concept analysis flowchart (Walker & Avant Method).

To ensure the reliability of the review, an independent peer review was developed to select the studies (AMPG, RAC, GML, and EIC). Initially, the titles and abstracts of the studies were read, selecting them for full reading according to the eligibility criteria. In case of disagreement, an evaluation was requested from the other pair. However, if the abstract reading did not provide enough information, each of the researchers proceeded to read the entire article independently. Subsequently, disagreements were resolved through joint discussions and consensus (AMPF, RAC, GML, and EIC). To systematically extract and organize the definition, attributes, antecedents, and consequences of care dependency in older adults, we applied a structured reading guide, supported by a logical and evidence‐based decision‐making algorithm in five stages (Figure 1).

This tool allowed researchers to identify and categorize recurring concepts within the literature. Following this extraction, a group discussion session was held in January 2023 to critically review the preliminary categories, identify patterns, and ensure a consistent and conceptually sound classification.

For the final selection of the study sample, the PRISMA statement guide was used, and the review results were represented based on it [27].

### 2.5 Quality Evaluation

For the study of methodological quality and evidence level of the studies that made up the final sample, the Johns Hopkins Nursing Evidence‐Based Practice Guide was used [28].

## 3 Results

### 3.1 Study Characteristics

**FIGURE 2:**
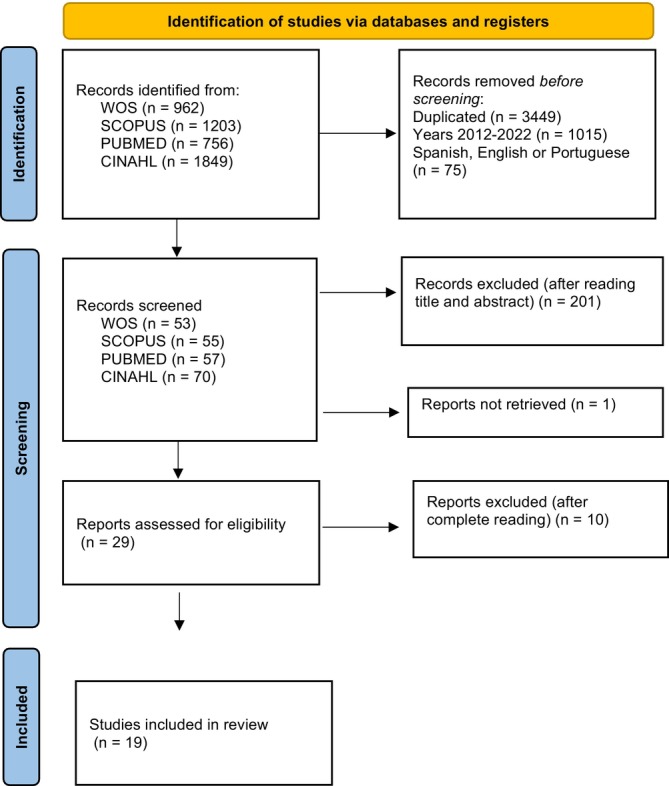
PRISMA flow diagram.

The database search yielded a total of 4531 articles. 1883 duplicate or invalid articles were deleted, and 2648 articles were reviewed. The exhaustive reading of all of them, applying the inclusion and exclusion criteria, and allowing the ethical and cross‐cultural analysis of the concept studied, resulted in a sample of 118 articles from the databases, and 33 new secondary review articles were added, also applying the quality check. Finally, a total of 132 articles were discarded. The final sample of the study was 19 articles (Figure 2).

Then, the data extraction made up the final study sample is presented, based on its objective, design, definition of the concept, attributes, antecedents, consequences, and methodological quality (Table 1). The quality of the articles has been determined using the level of evidence and the Johns Hopkins Quality Guide. From highest to lowest quality: 2 quality II articles (1 IIB and 1 IIC), 14 quality III (6 IIIA, 4 IIIB, 4 IIIC), 1 quality IV (1 IVB), and 2 quality V (1VA and 1VB).

**TABLE 1:** Summary table of included studies.

Author (year)	Aim	Design	Participants	Elements and attributes of the concept	Definition and antecedents	Consequence	Level of evidence
Country of origin							
Boggatz et al. (2007) Germany	To identify the meaning of care dependency that can be shared by both caregivers and care recipients	Concept analysis Walker and Avant's method for concept analysis.	The Medline, CINAHL, and Cochrane databases were searched for the period 1996–2006	Vulnerability (situation of disadvantage concerning the other/other people)	The person loses the ability to perform BADL	Secondary and subjective need for support in the domain of care. Need help from others.	III‐B
Smalbrugge M., Jongenelis, L., et al. (2007) Netherlands	To determine prevalence, course, correlates, recognition and treatment of pain among Dutch NH patients, and to make a comparison with international data	Observational cohort study	350 elderly NH patients from 14 Dutch NH's.	Physical (pain) and mental suffering. Loss of quality of life.	Self‐care capacity deficit	Provision of extensive and continuous care. Institutionalisation.	III‐B
Eriksson, M., y Andershed, B. (2008) Sweden	To increase the understanding of the meaning of being dependent on care from other people, the term caregiver is used both for family and for professional caregivers	Phenomenological hermeneutic method.	13 patients.	Perceived degradation. The feeling of burden and shame. Adaptive process. Estrangement from their own body/loss of identity. Change in the relationship with oneself and with others.	The person is not able to take care of himself.	Need for care by a caregiver, who can be a family member or a professional.	III‐C
Abad‐Corpa et al. (2012) England	To understand the process of adaptation to dependency in older adults and their families	Interpretive synthesis of qualitative studies	2164 Potentially relevant papers.	Subordination. Life change/biographical rupture. The feeling of burden. Adaptive process. Resignation. Mental or spiritual suffering. Vulnerability.	Loss of autonomy for BADL, influenced by age and chronic diseases.	Need for socio‐sanitary care.	III‐C
Abma T, Bruijn A, Kardol T, Schols J, Widdershoven G. (2012) Netherlands	Contribute to the current discussion on the autonomy of institutionalised older people.	Several in‐depth interviews	One participant. A ninety‐two‐year‐old man	Low self‐esteem. Identity loss. Lack of dignity.	Health declines that produce a loss of autonomy in terms of independence	Need for care. Institutionalisation, which at the same time produces an increase in dependency.	V‐B
Svanstrom, R., Sundler, A., (2013) Sweden	This study aimed to elucidate and gain a deeper understanding of elderly patients' experiences of suffering in community care in nursing homes and home care services.	Hermeneutical approach, Phenomenological interviews and conversations	The data material consisted of qualitative interviews and conversations with 25 participants	Insecurity. Loneliness. Loss of identity. Vulnerability. Risk of physical and mental suffering.	A chronic disease that generates loss of autonomy for BADL	Requires care at home or institutionalisation. Need of others.	III‐C
Risco, Era, Jolley, et al. (2015) Eight European countries	To investigate the association between specific categories of physical dependency and the presence of neuropsychiatric symptoms in people with dementia admitted to a long‐term care institution	a prospective observational cohort study	116 recently institutionalised dementia sufferers and 949 people with dementia still living at home	Mental health problems	Impaired cognition and physical functioning, or progression of degenerative diseases	Need for care by caregivers at home or institutionalisation	III‐A
Piredda, Matarese, Mastroianni, et al. (2015) Italy	This study aimed to describe adults. Patients' experience of nursing care dependence	Meta‐synthesis was conducted to integrate qualitatively	Findings from 18 studies published through December 2014	Estrangement from their own body/loss of identity. Experience of humiliation and insecurity. Interaction/impact on the relationship with the caregiver. Mental suffering. Nurse–patient relationship experienced as a good or bad experience	A chronic disease that causes decreased autonomy for BADL	Need for nursing care. Increased mortality	II‐B
Tuominen L, Leino‐Kilpi H, Suhonen R (2016) Finland	Identify experiences (realisation, facilitators, and barriers) of the free will of older people in nursing homes to improve the ethical quality of care	semi‐structured interviews	15 cognitively intact people over 65 years of age in 4 nursing homes	Loss of freedom of decision or choice and self‐determination	Loss of functional ability due to age	Need for care in institutions or at home. In both cases, the need for order, safety, and efficiency prevails over the decisions and wishes of the elderly.	III‐A
Fjordside S, Morville A. (2016) Denmark	To review the literature on how older people perceive opportunities and limitations regarding participation in autonomous decision‐making about their daily care in their own homes.	Literature review.	The review includes 12 publications from 2009 to 2014	Loss of the perception of control over their health, of individual freedom or self‐determination (they choose for themselves). Vulnerability. Interrelationship between caretaker‐caregiver.	Loss of functional autonomy	Need for care.	V‐A
Oosterveld‐Vlug MG, de Vet HC, Pasman HR, van Gennip IE, Willems DL, Onwuteaka‐Philipsen BD. (2016) Netherlands	To explore what characteristics of nursing home residents are related to factors that influence their dignity	Questionnaires containing the Measurement Instrument for Dignity Amsterdam e for Long‐Term Care Facilities (MIDAM‐LTC) were administered	95 nursing home residents, residing in the general medical wards of six nursing homes	Loss of dignity. Feelings of depression, and hopelessness. Desire to die	Ageing, functional, or cognitive decline	Need for care. Institutionalisation.	III‐B
Lohne V, Hoy B, Lillesto B, Saeteren B, Heggestad AKT, Aasgaard T, et al. (2017) Norway, Sweden, Finland	Explore how dignity is conceptualised, encouraged and supported by nursing home staff.	Hermeneutics (Focus Groups)	40 members of health personnel (mostly nurses) and non‐health personnel from 6 nursing homes.	Threatened dignity. Lack of respect for individuality, their wishes, and the principle of autonomy. Person‐caregiver interaction. Vulnerability. Suffering. Frustration. Feelings of impotence. Lack of privacy.	Ageing. Physical deterioration.	Need for care.	III‐A
Fahey A, Ní Chaoimh D, Mulkerrin GR, Mulkerrin EC, O'Keeffe ST. (2017) Ireland	To investigate how participants balance different attributes: place of residence (own home or nursing home), risk of harm (high, moderate, or low probability of a potentially harmful incident within a year), burden on the family (a lot, some, a little), life expectancy (1, 2, 3 years)	Community‐dwelling older hospital patients were asked, and Conjoint Analysis.	102 inpatients aged 65–80 years	Feeling of burden	Progression of physical or cognitive impairment that makes it difficult to live alone. Loss of autonomy for BADL	Institutionalisation: Aversion to entering a nursing home	III‐B
Domínguez González et al. (2018) Spain	To consider decisively the motivations, capacities and the possibilities of people integrating them in the management of dependency	Deductive methodology: a group of experts and a review of the literature	Expert groups and literature review	Loss of self‐determination. Loss of respect for needs and wishes	A condition inherent in human nature that materialises throughout life to different degrees. Functional disability for the development of BADL.	Need for help from another person	IV‐B
Caspari et al. (2018) Norway	To present results from interviews of older people living in nursing homes, on how they experience freedom	Hermeneutic, with qualitative research interviews	28 residents living in nursing homes in Denmark, Sweden, and Norway.	Loss of self‐determination, freedom, and abandonment of routines. Mental suffering.	Impairment of health and senses. The person is not capable of taking care of himself and cannot carry out BADL autonomously.	Need for care by another person at home, or in an institution.	III‐C
Henskens et al. (2019) Netherlands	To examine the contribution of multiple predictors in predicting care dependency	Longitudinal descriptive study	85 nursing home residents	Loss of quality of life	Functional incapacity, dementia, and other health problems (i.e., malnutrition, pressure ulcers, incontinence, falls) Progressive deterioration in activities of daily living (ADL)	Need for care	II‐C
Seidlein AH, Buchholz I, Buchholz M, Salloch S (2019) Germany	To explore the points of view of dependent elderly people, informal caregivers, and professional caregivers about their experiences with “care overload”.	Semi‐structured interviews	Older people in need of long‐term home care [10], informal carers [8] and professional carers [10]	Loss of dignity, suffering, and desire to hasten death. Loss of self‐sufficiency, lifestyle, and autonomy. Isolation. Feelings of burden. Marginalisation and social oblivion.	Ongoing decrease of functional capacity, which limits their mobility	Permanent dependency on the help of others, either at home or in an institution. The help of others	III‐A
Boga & Saltan (2020) Turkey	To examine the dependency levels of older adults living in nursing homes and home environments in their daily life activities, and the relationship involving pain, sleep, depression, and mental status affecting these levels	A comparative‐descriptive and cross‐sectional study	185 older adults living in a nursing home, and those in home environments.	Pain, biological rupture/life change, sleep disorders, depression, loss of quality of life.	Loss of functional capacity, of independence to perform BADL	Need for care by another person at home, or in an institution. Increased risk of mortality and cost of health care	III‐A
Muldrew DHL, Kaasalainen S, McLaughlin D, Brazil K (2020) UK, Canada	Identify the type of ethical issues and associated level of distress experienced by nurses providing palliative care in nursing homes.	A cross‐sectional study	123 nurses from 21 nursing homes in the UK and Canada. One hundred and twenty‐three nurses located in 21 nursing homes across the UK and Canada completed the EPiCNH instrument.	Loss of personal freedom. Complex care needs.	Ageing	Care needs	III‐A

### 3.2 Definition and Use of the Concept

To systematically extract and organise definitions, attributes, antecedents, and consequences of care dependency in older adults, from an ethical and transpersonal perspective, a concept analysis procedure was conducted using Walker and Avant's framework [24]. The guiding question for this analysis was: *What is the concept, the attributes or characteristics, the antecedents, and consequences of care dependency in older people from an ethical and transpersonal perspective?* Furthermore, we applied a structured reading guide, supported by a logical and evidence‐based decision‐making algorithm in five stages (Figure 1). This approach ensured analytical rigor and traceability in the identification and classification of elements.

#### 3.2.1 Care Dependency Concept

The concept refers to the phenomenon, idea, or construct that is intended to be clarified. Its purpose is to clearly define what the concept under analysis is and why it is relevant. In this study, the concept in question is care dependency.

Care dependency in older adults is a multifactorial condition characterised by the progressive loss of autonomy in performing basic and instrumental activities of daily living, influenced by physical, cognitive, emotional, and social deterioration [1, 4, 29, 30, 31, 32]. This condition is not solely determined by biomedical factors but is also shaped by personal, relational, and existential dimensions [4, 33, 34, 35].

It entails the need for assistance from others to ensure the individual's well‐being and safety, leading to the development of interpersonal care relationships. These relationships often involve a reconfiguration of identity, dignity, and vital roles, potentially resulting in emotional suffering and difficulties in coping [33, 35, 36, 37, 38]. From an ethical and transpersonal perspective, care dependency is not merely a state of deficit but rather a human experience that demands presence, respect, and recognition of personal vulnerability and dignity [4, 33, 34, 35, 39, 40].

#### 3.2.2 Definition of the Attributes

Attributes are the essential characteristics or features that consistently appear in literature and are fundamental to the concept's existence. They represent the core dimensions of care dependency, in this case from an ethical and transpersonal perspective, and serve to differentiate it from other related concepts. Identifying these attributes enables us to determine when and how the concept manifests. Following the process described in Figure 1, attributes were identified during *Step 3: Preliminary Classification*, considering only those elements that directly described the concept of care dependency from an ethical and transpersonal perspective. These were subsequently validated through the model, borderline, and contrary cases to distinguish essential attributes from contextual ones. This procedure led to the identification of four core attributes, closely supported by literature, relating to loss or erosion of: autonomy and personal dignity, identity and self‐image, biographical rupture, and coping:

1. Loss of autonomy
  It encompasses the reduction or loss of self‐determination and freedom of choice [33, 37, 41], understood as a significant limitation in decision‐making, which includes the lack of recognition and respect for such needs, will, and wishes by others [20, 29, 32, 34, 38, 42, 43]. This significant limitation in the ability to make decisions and act following one's own will arises as a result of functional and/or cognitive restrictions.
2. Erosion identity and self‐image
  Progressive loss or significant alteration of personal identity, understood as the change or weakening of the roles, values, and beliefs that define one's sense of self [21, 30, 33, 35, 37, 38, 43, 44].
  This is accompanied by an alteration of self‐image, understood as the physical and sensory perception of oneself, which is affected by functional decline, such as the need for assistance with intimate activities and the loss of control over one's appearance [21, 33, 37, 40].
3. Biographical rupture
  Significant disruption in the continuity of life history, characterised by a break in life continuity and an alteration of personal narrative [31, 34, 42], marked by drastic changes in routines, roles, and relationships [4, 29, 42].
4. Negative Coping
  It refers to the use of ineffective or counterproductive cognitive, emotional, and/or behavioural strategies to manage the situation of dependency, which fail to resolve the problem and may increase physical, psychological, or social distress. These responses include passive resignation, pessimistic or catastrophic thoughts, abandonment of self‐care, social withdrawal, and the desire to die, among others [4, 35, 38, 43, 44].

#### 3.2.3 Illustrative Case Examples

Following the Walker and Avant concept analysis method, three theoretical cases were constructed to validate the defining attributes of care dependency as viewed through an ethical and transpersonal lens in older adults.

##### 3.2.3.1 Model Case and Analysis

Mrs. García, an 80‐year‐old woman, lives alone in a third‐floor apartment without an elevator. She suffers from hypertension, DM II, hypercholesterolemia, osteoporosis with persistent low back pain, gait instability, mixed urinary incontinence, a Body Mass Index (BMI) of 32, INICIARE of 80 [45], and GDS 2 according to the Reisberg Global Deterioration Scale [46], with a prescription of more than 9 drugs including insulin.

The following analysis outlines the main attributes of care dependency identified in her situation:

- Loss of autonomy: Due to cognitive impairment and reduced mobility, she has difficulty performing basic activities of daily living such as bathing, dressing, eating, and managing her medication. She previously received help from her husband, but since his passing, she depends on her son and a caregiver, whom he selected without her approval, leading her to feel that her needs, wishes, and will are not taken into account. Accepting her son's assistance with intimate tasks such as personal hygiene or dressing is difficult for her, generating feelings of embarrassment and of being a burden to him. This situation causes her a profound sense of loss of freedom and respect. Her son is now also considering placing her in a nursing home.
- Erosion of identity and self‐image: The change in her role and dependence on her son has affected her self‐image, shifting from perceiving herself as an independent woman to viewing herself as dependent and limited.
- Biographical rupture and vital breakdown: The loss of her husband and the reduction in her social and recreational life due to her limitations have fragmented her life story. Her upcoming admission to a nursing home—something she never wished for—represents a definitive break with her home, her neighbourhood, and her friendships.
- Negative coping responses: Although she understands her son's situation and the need for change, she experiences sadness and resignation, alternating with periods of apathy. At times, she expresses that she would prefer not to go on living under these conditions.

She comes from a cultural background where older adults are expected to participate actively in decisions about their care, and this makes the lack of consultation in choosing her caregiver particularly distressing. Reduced participation in community and family roles has weakened her sense of identity, while moments when her son or caregiver listens to her stories or incorporates her preferred routines provide relief, illustrating how transpersonal care practices can help preserve dignity and meaning.

##### 3.2.3.2 Borderline Case and Analysis

Mrs. Martínez, an 85‐year‐old woman, has a BMI of 30 and suffers from hypertension, hypercholesterolemia, recurrent urinary tract infection, and insulin‐dependent type II diabetes mellitus. She lives alone on the third floor without an elevator since the death of her husband a year ago, and she expresses feelings of sadness and loneliness. Her sister and her friends live nearby, but she cannot go down the stairs well, so she is limited in going for a walk with them and doing the exercise recommended for her diabetes. The following analysis identifies the main attributes of care dependency present in her situation:

- Partial loss of autonomy: She receives weekly assistance from her daughter, who lives far away, for grocery shopping (mainly pre‐cooked food), some household chores, and hair washing. While she manages most basic activities independently, her physical and mobility limitations constrain her independence and limit her decision‐making capacity in certain daily matters.
- Emerging erosion of identity and self‐image: The reduction in her social activity and her partial reliance on her daughter's help have altered her self‐perception, although she maintains the will to live in her own home and to actively engage in her self‐care.
- Moderate biographical rupture: The loss of her husband and the reduction in her social life have modified her routines and sense of life continuity, yet she retains emotional bonds and motivation to sustain them.
- Positive adaptive responses: Her daughter, concerned for her well‐being, is looking for a ground‐floor apartment close to family and friends, which is expected to improve her quality of life and preserve her independence, thus avoiding nursing home placement and respecting her wishes.

Although her daughter recognizes the potential benefits of a nursing home in terms of safety and access to formal care, she is also aware that her mother's cultural values prioritize aging in place and maintaining community connections.

##### 3.2.3.3 Contrary Case

Mr. Navarro is 79 years old and lives alone in his home, located on the third floor with an elevator. He has type II DM that he controls with exercise and diet (BMI 22), grade II hearing loss, 3.5 diopter presbyopia; he has been a hip prosthesis carrier for 10 years and prostatitis under treatment. He has an active life, walks his dog several times a day, swims 3 times a week, and in the afternoon, he attends the Civic Center in his area where he does activities with his friends. He visits the family regularly and, although he could do the housework, he prefers to pay for the services of a housekeeper to have more free time. The following analysis identifies the main attributes of care dependency present in his situation:

- Loss of autonomy: Not present. Maintains full functional capacity and makes his own decisions without relying on others for daily activities.
- Erosion of identity and self‐image: Not present. Maintains a positive self‐image and an active identity consistent with his independent lifestyle.
- Biographical rupture/life break: Not present. His life routine is stable, with no events that have significantly disrupted his personal or social trajectory.
- Negative coping: Not present. Demonstrates good coping abilities, motivation, and emotional stability.

The analysis of the three cases illustrates a continuum from full care dependency to complete autonomy, clarifying the scope and applicability of the defining attributes proposed in this study.

The model case (*Mrs*. *García*) represents a comprehensive scenario of care dependency. All four attributes are clearly present: loss of autonomy and dignity, through limitations that prevent her from making decisions about her own care and lifestyle; erosion of identity and self‐image, as her dependency alters her self‐perception from independence to burden; biographical rupture, marked by the loss of her spouse, social disconnection, and change in living environment; and negative responses, including sadness, resignation, and expressions of not wanting to live under current conditions. This profile embodies the ethical and transpersonal dimensions of care dependency, showing the interplay between functional limitations, relational changes, and emotional consequences.

The borderline case (*Mrs*. *Martínez*) reflects a situation of moderate dependency and vulnerability. Although she experiences social isolation, some role adjustments, and limited autonomy due to her physical constraints, the loss of dignity is not as pronounced, and her coping remains largely positive, sustained by the prospect of moving to an environment that could restore independence. Negative responses are minimal, and the transition planned by her daughter may prevent progression towards full dependency.

In contrast, the contrary case (*Mr Navarro*) demonstrates the complete absence of the defining attributes. He maintains autonomy, dignity, and active social engagement, with functional adaptations that allow him to lead a fulfilling and independent life. His situation highlights that the presence of chronic conditions or ageing‐related changes does not necessarily equate to care dependency when self‐determination and functional capacity are preserved.

The three cases also reveal that cultural context shapes the expression and impact of care dependency attributes. For instance, values related to ageing in place, family involvement, and community participation influence how older adults perceive loss of autonomy or identity. In the model case, limited consultation and restrictions on customary roles intensified the sense of dependency; in the borderline case, family dialogue and respect for preferences helped maintain dignity; and in the contrary case, cultural expectations had little impact due to the absence of dependency attributes. Through these scenarios, transpersonal care, characterised by mutual respect, attention to life story, and alignment with personal values, emerges as a key element in either mitigating or preventing the negative consequences of care dependency.

Collectively, these cases enhance the conceptual clarity of care dependency in older adults from an ethical and transpersonal perspective and support the proposed attributes as valid indicators for understanding and assessing the phenomenon.

### 3.3 Antecedents

Care dependency in older people arises from a series of progressive and cumulative factors that diminish autonomy and increase vulnerability. One of the primary antecedents is the ageing process, which leads to a gradual decline in both functional and cognitive capacities [1, 33, 42]. Functional deterioration is evident in the inability to perform basic activities of daily living, while cognitive decline affects memory, decision‐making, and orientation [4, 29, 34, 37, 40].

The presence of chronic and degenerative diseases—such as diabetes, osteoarticular disorders, and neurological conditions—further contributes to the onset of care dependency [1, 33, 39, 47, 48]. These clinical and physiological factors are exacerbated by the progressive loss of autonomy, understood as a diminished capacity for self‐care and decision‐making regarding health [33, 35, 37].

From a psychosocial perspective, a lack of social support [4, 30, 32] and emotional vulnerability—characterised by hopelessness, sadness, and low self‐esteem—are critical enabling factors [31, 34, 35, 41]. Additionally, cultural and institutional vulnerability, stemming from structural inequalities, social exclusion, or limited access to quality care, further constrains the potential for dignified ageing [1, 21, 29, 44].

### 3.4 Consequences

Once care dependency, from an ethical and transpersonal perspective, is established, its effects manifest across multiple dimensions. According to each of the attributes (1. Loss of autonomy, 2. Erosion of identity and self‐image, 3. Biographical rupture, and 4. Negative coping): It is reflected in a decrease in quality of life and lifestyle [29, 31, 38, 47], accompanied by feelings of disrespect, loss of freedom, and threats to personal dignity [33, 35, 38, 43, 44]. In this context, individuals are vulnerable and may experience humiliation, degradation, shame, subordination, and loss of privacy [1, 4, 21, 29, 32, 34, 35, 37, 40, 48]. These experiences often lead to emotional insecurity and the risk or actual occurrence of mental and physical suffering, such as pain [4, 29, 31, 33, 37, 40, 41, 44, 49].

The feeling of loss of belonging and of not recognizing oneself may trigger a sense of detachment from one's own body, along with decreased self‐esteem and feelings of burden and hopelessness, which erode one's sense of personal worth [21, 30, 33, 37, 43, 44].

This rupture involves the loss of projects, goals, and meaningful activities, as well as a reduction in social and recreational life and the sense of belonging [21, 35, 38].

In the context of care dependency, such coping may intensify the perception of loss of control and dignity and contribute to the onset of depression, frustration, and helplessness [4, 35, 38, 43, 44].

These repercussions are not limited to the dependent person but also significantly affect caregivers, creating physical, emotional, and social strain for both family members and professional care providers [21, 37, 38, 40]. In many cases, this situation progresses towards institutionalisation, often against the person's wishes, which further diminishes control over their environment [30, 41, 44].

**FIGURE 3:**
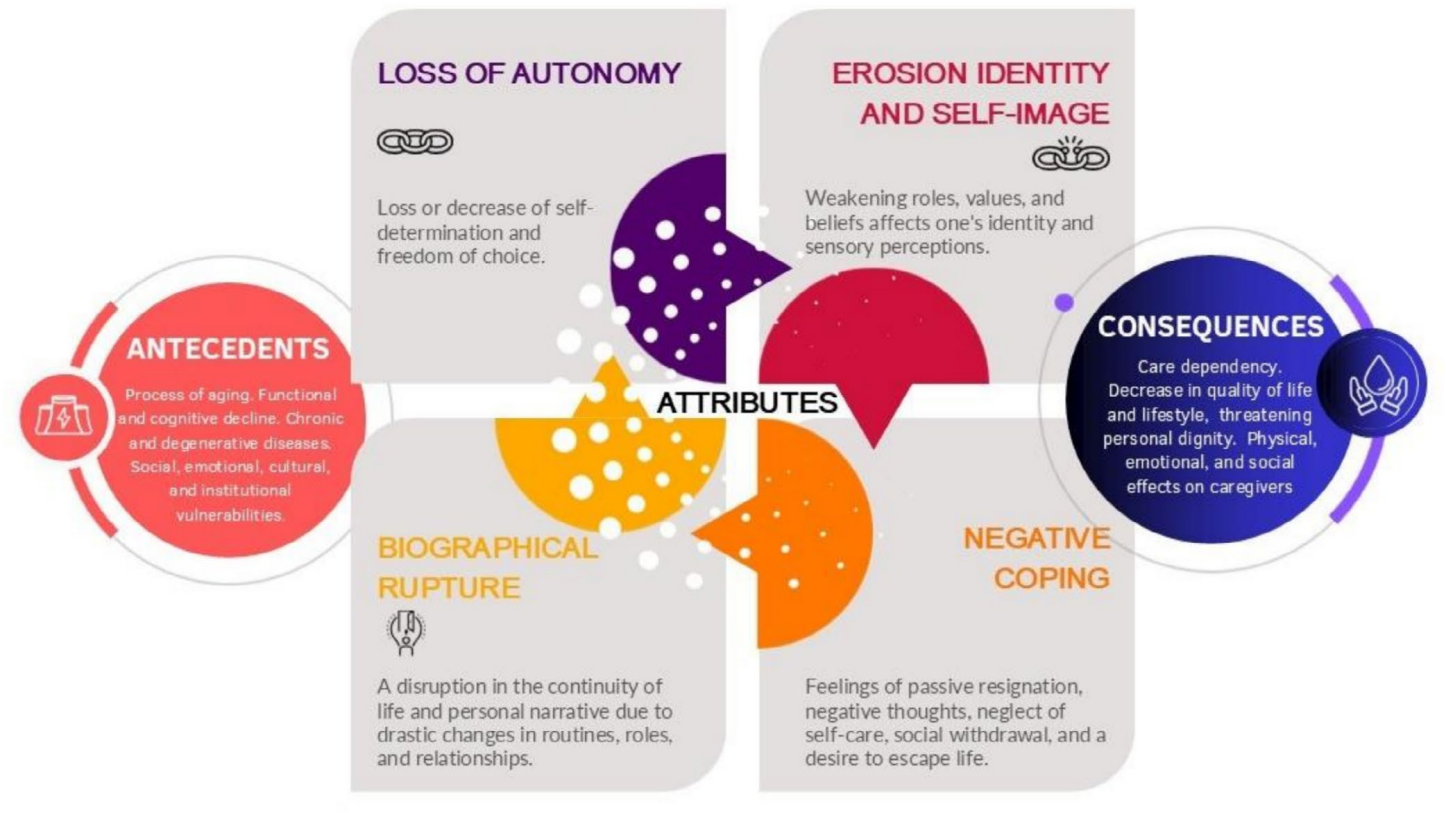
Conceptual map of care dependency in older people from an ethical and transpersonal perspective.

A conceptual map (Figure 3), which includes the concept, antecedents, and consequences of care dependency in older people, from an ethical and transpersonal perspective, is described in Figure 3.

### 3.5 Empirical References

According to Step 8 of Walker and Avant's concept analysis method, operationalising the concept of care dependency and linking its defining attributes to observable indicators in clinical practice requires the identification of validated assessment tools. These empirical references enable the objective measurement of the presence and degree of dependency, thereby reinforcing the theoretical foundation of the concept and supporting its application in care settings.

There are some validated tools to measure the presence of dependency in care. Regarding the person's ability to perform basic activities of daily living, the Barthel index was one of the first to be developed [50]. It consists of 10 items that assess the ability to perform certain activities without help. The score ranges from 0 (completely dependent) to 100 (completely independent), and the response in each category ranges from 2 to 4 alternatives, with intervals of 5 points. In its initial version, it obtained an alpha internal consistency coefficient of 0.87 to 0.9 [50].

Regarding the ability to perform instrumental activities of daily living, there is the Lawton and Brody scale [51]. This scale evaluates eight domains, each of which is assigned a numerical value of 1 (independent) or 0 (dependent). The total score is obtained after the sum of all the items and reflects the degree of independence of the evaluated subject (0 points, as maximum dependence; 8 points, total independence). In the initial version, it obtained an alpha internal consistency value of 0.88 (range, 0.80–0.99) [51].

The Care Dependency Scale deserves special mention, conceptually based on the level of dependency of the person according to their care needs, considering not only physical but also psychosocial aspects [52]. Based on Virginia Henderson's conceptual model, it consists of 15 items and a five‐point Likert‐type scale. Scores range from 15 to 75 points per patient, with lower values indicating higher levels of care dependency. In the initial version, it obtained an alpha internal consistency value of 0.88 [52].

A more recent scale, based on the nursing terminology of the International Classification of Outcomes (NOC), INICIARE Scale, proposes not only the evaluation of dependency in care but also a classification tool for it at levels [45]. In its short version, it consists of 26 items and 5 factors, with Cronbach reliability alpha values ranging between 0.90 and 0.94. For each item, the range of scores is from 0 to 5, where 5 reflects the most desirable state of the patient and 1 is the least desirable. The total score ranges from 26 points (indicating the highest level of dependency) to 130 points, indicating independence [45].

## 4 Discussion

This concept analysis, conducted using Walker and Avant's method [24], identified four defining attributes of care dependency—loss of autonomy, erosion of identity and self‐image, biographical rupture, and negative coping—together with antecedents and consequences that deepen understanding of the phenomenon. Care dependency is not simply a manifestation of physical or cognitive decline, but a multidimensional condition shaped by interpersonal relationships, socio‐cultural contexts, and ethical considerations. Mapping attributes to consequences enhances clinical applicability: loss of autonomy often leads to loss of freedom and respect; erosion of identity to isolation and diminished self‐esteem; biographical rupture to social disconnection and loss of meaning; and negative coping to accelerated decline and emotional distress. These interconnections highlight targets for intervention and support the design of holistic, person‐centred care strategies that preserve dignity and self‐determination [53, 54, 55, 56, 57].

The three theoretical cases illustrate a continuum from full dependency to complete autonomy. The model case shows all attributes with severe ethical and emotional impacts; the borderline case reflects partial dependency with potential for positive adaptation; and the contrary case reinforces that ageing or chronic illness does not inevitably result in care dependency. Collectively, they confirm the conceptual validity of the attributes and show how context and culture shape their expression.

Dependency should be understood as a fundamental human condition linked to vulnerability and intersubjectivity [58]. Mutual dependency underpins the caring encounter, where the transpersonal dimension, rooted in presence, empathy, and reciprocity, becomes central [59]. Autonomy, as a relational right, should be promoted even under vulnerable conditions, requiring communication, shared decision‐making, and cultural respect [17, 18, 32, 42, 60, 61, 62, 63, 64]. This approach mitigates paternalistic care and aligns with bioethical principles, ensuring integrity and human rights are upheld.

Care dependency often arises from age‐related conditions or crises affecting both the individual and family dynamics, altering roles and intimacy [65]. These transformations should be interpreted through transpersonal care, where the professional–patient relationship transcends technical intervention to become an intersubjective space of meaning [66]. Nursing theories such as Watson's Human Caring and Eriksson's Caritative Caring offer frameworks for integrating ethical, relational, and spiritual dimensions into practice [67, 68].

Institutionalisation, while sometimes beneficial, frequently exacerbates dependency through loss of freedom, identity, and social connection [1, 29, 31, 32, 33, 34, 35, 69]. Home‐based care can better maintain autonomy and familiarity, though LTC facilities may offer structured support and social engagement when care is person‐centred [70, 71, 72, 73, 74]. The setting in which care occurs significantly shapes how attributes manifest, requiring interventions tailored to context.

Cultural variability also influences how dependency is experienced: collectivist contexts may buffer biographical rupture by maintaining roles, while individualistic cultures may perceive loss of autonomy as a greater threat to dignity [75, 76]. Transpersonal practices, emphasizing the person's life story, values, and cultural background, can bridge these differences, reducing isolation and loss of meaning [77, 78]. The case examples explicitly incorporate these perspectives, showing their practical relevance.

Ultimately, care dependency in older adults is an ethically situated phenomenon. Applying a transpersonal lens in nursing entails recognizing life history, values, and cultural context as integral to care decisions, operationalized through presence, active listening, and shared decision‐making. Educationally, it supports integrating transpersonal and cultural competence into nursing curricula; at the policy level, it calls for guidelines embedding cultural assessment and personhood‐focused interventions.

As a concept analysis rather than empirical research, the study's contribution lies in its conceptual transferability and theoretical applicability. Limitations include variability in definitions across studies, differences in care settings, and the exclusion of overlapping terms such as frailty or vulnerability. Language restrictions and the omission of grey literature may have excluded relevant perspectives, and limiting the review to publications up to a certain year may have missed recent developments. Variations in definitions and methodological approaches in primary studies could also have influenced synthesis coherence.

## 5 Conclusions

Care dependency is revealed not as a purely functional or cognitive limitation, but as a multidimensional phenomenon shaped by interpersonal relationships, socio‐cultural contexts, and ethical considerations. The analysis underscores the centrality of recognizing relational asymmetry, vulnerability, and perceived need as defining attributes, while also emphasizing the ethical tensions inherent in balancing respect for autonomy with the principle of beneficence.

This concept analysis refines the attributes, antecedents, and consequences of care dependency within an explicitly transpersonal and culturally sensitive framework. The model, borderline, and contrary cases illustrate how these elements manifest across a continuum of dependency, and how transpersonal care can mitigate negative consequences by fostering dignity, meaning, and connection, while supporting ethically sound decision‐making that honors the person's values and life story.

These findings refer to the proposed conceptual framework, which offers a foundation for guiding clinical assessment, communication strategies, and policy development. Future research should explore its applicability through qualitative inquiry, such as interviews or ethnographic studies, to capture lived experiences in diverse care settings. Such work would support the translation of this framework into actionable, ethically grounded tools and interventions, enabling nurses to deliver culturally attuned, transpersonal care in daily practice.

## Author Contributions

Ana‐María Porcel‐Gálvez, Enrique‐Octavio Iñiguez‐Castro, Gloria Martínez‐Lacovic, and Regina Allande‐Cussó made substantial contributions to conception and design, or acquisition of data, or analysis and interpretation of data. Ana‐María Porcel‐Gálvez, Enrique‐Octavio Iñiguez‐Castro, Gloria Martínez‐Lacovic, and Regina Allande‐Cussó involved in drafting the manuscript or revising it critically for important intellectual content. Ana‐María Porcel‐Gálvez and Regina Allande‐Cussó Given final approval of the version to be published. Each author should have participated sufficiently in the work to take public responsibility for appropriate portions of the content. Ana‐María Porcel‐Gálvez and Regina Allande‐Cussó agreed to be accountable for all aspects of the work in ensuring that questions related to the accuracy or integrity of any part of the work are appropriately investigated and resolved.

## Ethics Statement

The authors have nothing to report.

## Conflicts of Interest

The authors declare no conflicts of interest.

## Data Availability

The data that support the findings of this study are available from the corresponding author upon reasonable request.
